# Will Women Diagnosed with Breast Cancer Provide Biological Samples for Research Purposes?

**DOI:** 10.1371/journal.pone.0127994

**Published:** 2015-06-10

**Authors:** Shelley A. Harris, Beatrice A. Boucher, Michelle Cotterchio

**Affiliations:** 1 Prevention and Cancer Control, Cancer Care Ontario, Toronto, Ontario, M5G 2L7, Canada; 2 Dalla Lana School of Public Health, University of Toronto, Toronto, Ontario, M5T 3M7, Canada; 3 Occupational Cancer Research Centre, Toronto, Ontario, M5G 1X3, Canada; 4 Department of Nutritional Sciences, University of Toronto, Toronto, Ontario, M5S 3E2, Canada; University of Campinas, BRAZIL

## Abstract

**Background:**

Little is known about the response rates for biological sample donation and attitudes towards control recruitment, especially in younger women. The goals of this pilot study were to determine in women recently diagnosed with breast cancer, the proportion of cases willing to provide biological samples and for purposes of control recruitment, contact information for friends or colleagues.

**Methods:**

A population-based sample of breast cancer cases (n = 417, 25-74 years) was recruited from the Ontario Cancer Registry in 2010 and self-administered questionnaires were completed to determine willingness to provide samples (spot or 24-hr urine, saliva, blood) and contact information for friends/colleagues for control recruitment. Using Χ^2^ analyses of contingency tables we evaluated if these proportions varied by age group (<45 and 45+) and other factors such as ethnicity, education, income, body mass index (BMI), smoking status and alcohol consumption.

**Results:**

Cases were willing to provide blood samples, by visiting a clinic (62%) or by having a nurse visit the home (61%). Moreover, they would provide saliva (73%), and morning or 24-hr urine samples (66% and 52%). Younger cases (≤45) were 3 times (OR) more likely more than older cases to agree to collect morning urine (95% CI: 1.15-8.35). Only 26% of cases indicated they would provide contact information of friends or work colleagues to act as controls. Educated cases were more likely to agree to provide samples, and cases who consumed alcohol were more willing to provide contact information. Ethnicity, income, BMI and smoking had little effect on response rates.

**Conclusions:**

Reasonable response rates for biological sample collection should be expected in future case controls studies in younger women, but other methods of control selection must be devised.

## Introduction

The increased use of biological samples to measure biomarkers of internal dose, early biological effects, and genetic susceptibility or modifiers has improved methods of exposure assessment in case control studies. The type of biological sample collected depends on the exposures/markers of interest and typically reflects longer term exposures; for example saliva or blood samples for genetic mutations/variants or markers of DNA damage, serum for environmental contaminants or infection history, and hair or toenails for metals exposures. Urine samples may be collected, where excreted compounds typically reflect more recent exposures (i.e., phytoestrogens, phthalates). We and others have gained experience collecting urine and other biological samples in large prospective population-based cohort studies of healthy individuals [1–5]. However, many case control studies of cancer using biological samples have focused on genetic determinants, the bulk of our experience is with the collection of saliva and blood samples, including studies conducted in Ontario, Canada [6,7].

In case control studies, it is important that response rates are maximized and that systematic bias in the provision of questionnaire information or biological samples does not occur. Thus, in the design phase of any study, an understanding of the expected response rates and how these might be affected by important confounding factors or subject inclusion criteria is needed. If confounding variables influence response rates or influence them differently by case control status, biased estimates of risk may result. In addition, if these factors influence response overall, certain group of individuals may be underrepresented in population based studies and this results in reduced generalizability.

Overall response rates for the provision of biological samples in previous population-based cancer studies have been reasonable [8–12], but typically minimal information is reported on how these response or participation rates vary by demographic characteristics, case or control status, or the type of cancer or disease under investigation. In a review of 365 epidemiologic studies published during 2003, Morton et al., [13] reported that participation rates declined during the 2007 to 2003 study period for all study designs, and that rates were reported in only 44% of case control studies. Over time, more studies collected biological samples but participation with biological sample collection was only reported in 27% of the studies overall and in 13% of the case-control studies. The authors emphasized the need to consistently report participation rates in all studies. Furthermore, there are few published studies reporting how participation rates, and those specifically for biological sample collection, are affected by potential confounding variables such as age, ethnicity, socioeconomic status, etc.

A cross-sectional study [14] was conducted in 2010 to assess phytoestrogen intake among breast cancer patients and to determine: 1) the proportion of breast cancer cases willing to provide biological samples (spot or 24 hour urine, saliva and blood samples), and/or contact information for friends or colleagues for future control recruitment, and 2) if these proportions varied by age group (<45 and 45+) and other factors such as ethnicity, education, income, body mass index (BMI), smoking status and alcohol consumption. These objectives were of interest as a larger case control study of young women with breast cancer and exposures to emerging environmental contaminants was planned, and it is now underway (the Ontario Environment and Health Study, OEHS)[15]. In the OEHS, women under the age of 45 (used as a proxy for menopausal status) are asked to provide questionnaire information (dietary and environmental) and biological samples (urine and blood) for biomarker analyses.

## Methods

Cases were Ontario women 25–74 years of age identified through the population-based Ontario Cancer Registry (OCR) ePATH system (electronic data transmission) as having a pathology confirmed first primary diagnosis of breast cancer between April and May 2010. Cases were sent a letter from the OCR notifying them they would be contacted to request their participation and were given a toll-free number to call (within 4 weeks) if they wished to opt out.

A self-administered questionnaire and cover letter were mailed to cases 2 months post-diagnosis, followed by post-card reminders and a second questionnaire package if necessary. The questionnaire included items to measure phytoestrogen food and supplement intake, treatment information and subject characteristics. Additional details on study design and methods are provided in Boucher et al. [14] A series of questions was asked on willingness to provide biological samples (blood, saliva and urine), and to provide names and contact information for female friends and/or colleagues of a similar age. The research was approved by the Research Ethics Board at the University of Toronto.

Questionnaire data were analyzed using SAS, Version 9.1. Proportions and 95% exact (Clopper-Pearson) binomial confidence intervals (CI) were estimated and Χ^2^ analyses of contingency tables were conducted to evaluate associations with categorical variables (i.e., age group, education, ethnicity, marital status, income, BMI, smoking, alcohol consumption) and the willingness to provide biological samples or contact information for friends,. Odds ratios (OR) and the associated 95% Wald CI were estimated using older cases as the referent group.

## Results

Of the 462 eligible cases identified by the OCR, 45 opted out of participation in research following contact by the OCR, leaving 417 cases that we were able to contact to request consent and participation in the study. Of the 417 cases contacted, there were 52 under the age of 45 and 365 aged 45+; 67% of these cases returned questionnaires. Characteristics of the responding breast cancer cases are presented in Table 1. The majority of cases were older than 50 years and most had graduated or had some college or university education. Of the total 278 cases, there were 33 cases (12%) under age of 45, and 245 (88%) who were 45 years of age and older. This age dichotomization was used in in the analyses reported in Table 2, where a description of participant willingness to provide samples and contact information for their friends is presented overall and stratified by age group. Cases were quite willing to provide blood samples, by either visiting a clinic (62%) or by having a nurse or technician visit their home (61%). Participants indicated they were most willing to provide saliva samples (73%). When stratified by age group, the younger cases (≤45) were consistently more likely to provide any of the biological samples, but differences between the two age groups were not statistically significant except for the morning urine sample. Younger cases were 3.1 times more likely to report they would collect a morning urine sample and courier it to our laboratory (95% CI: 1.15–8.35). Although younger cases were equally likely to indicate they would send in a saliva sample (84%) this was not a significantly higher positive response when compared to older women (71%).

**Table 1:** Descriptive characteristics of study participants (N = 278).

Characteristic	Subjects	
*Age (years)* ^*a*^	*N*	*%*
31 to 40	15	5%
41 to 50	56	20%
51 to 60	106	38%
61 to 71	101	36%
*Education completed*		
Some high school	29	11%
Graduated high school	61	22%
Some college or university	55	20%
Graduated college or university	128	47%
*Annual Income*		
< $ 40,000	41	15%
$ 40,000–59,999	41	15%
$ 60,000–99,999	61	22%
$100,000+	62	23%
Prefer not to answer	66	24%
*Ethnicity*		
*Caucasian*	227	82%
*Southeast Asians* ^*b*^	20	7%
*Other* ^*c*^	30	11%
*BMI* ^*d*^ *(kg/m* ^*2*^ *) one year ago*		
<18.5 (Underweight)	9	3%
18.5–24.9 (Normal weight)	111	42%
25.0–29.9 (Overweight)	89	33%
30+ (Obese)	58	22%
*Alcohol intake (drinks/week)* ^*e*^		
Never	116	49%
1–6	64	27%
≥7	56	24%
*Smoking status*		
Current	27	10%
Ever	101	37%
Never	146	53%

^*a*^ *Of the 278 cases there were 33 (12%) under the age of 45 and 245 (88%) 45 years of age and older*

^*b*^ Southeast Asian includes Japanese, Chinese

^c^ Other includes South Asian (eg. East India, Pakistan) and Black

^*d*^ BMI = body mass index

^e^Total drinks based on beer or hard cider (12 oz/350mL, wine (4 oz/120 mL), and sake, sherry, port, spirits, liqueurs, brandy or liquor (1 oz/30 mL)

**Table 2:** Overall and age related distribution of breast cancer cases willing to provide biological samples and contact information of friends and work colleagues.

Question	Age (years)									
	Overall			≤ 45			45+			Odds Ratio (95% CI)
	N	%	*95% CI* ^*a*^	N	%	*95% CI*	N	%	*95% CI*	
*Go to a local clinic to give a blood sample*										
Yes	161	62%	[56-68]	23	72%	[56-87]	138	61%	[55-67]	1.63 (0.72–3.68)
No	97	38%		9	28%		88	39%		
*Have a nurse or technician visit your home to take a blood sample*										
Yes	161	61%	[55-67]	20	63%	[46-79]	141	61%	[55-67]	1.06 (0.50–2.23)
No	102	39%		12	37%		90	39%		
*Provide a saliva sample and mail it back to the study laboratory*										
Yes	190	73%	[67-78]	27	84%	[72-97]	163	71%	[65-77]	2,22 (0.82–6.00)
No	72	27%		5	16%		67	29%		
*Collect a morning urine sample and courier it to our laboratory*										
Yes	170	66%	[60-72]	27	84%	[72-97]	143	64%	[57-70]	3.09 (1.15–8.35)
No	87	34%		5	16%		82	36%		
*Collect your urine throughout a whole day and courier it to our laboratory*										
Yes	130	52%	[45-58]	21	66%	[49-82]	109	50%	[43-56]	1.94 (0.90–4.22)
No	122	48%		11	34%		111	50%		
*Provide the names and contact information for women of a similar age*										
Yes	69	26%	[21-32]	11	35%	[19-52]	58	25%	[20-30]	1.64 (0.74–3.63)
No	193	74%		20	65%		173	75%		

^*a*^ CI = exact binomial confidence interval

Overall, only 26% of cases indicated they would be willing to provide contact information to approach friends or work colleagues to act as study controls. A slightly larger percentage of younger cases indicated they would provide this information (35% vs. 25% in 45+yrs). When age was assessed using 10 year age groups significant differences between groups were observed (Χ^2^ = 12.3, p = 0.007). Cases aged 41–50 years were most likely to agree to provide contact information for friends (43%). Younger and older cases were less likely to agree (range 17–20%).

In addition to the effects of age, the influence of education, ethnicity, income, BMI, smoking, and alcohol consumption on willingness to provide samples and contact information was assessed. Significant differences were not observed by smoking status or ethnic categories, but the majority of the sample was white (82%), with only 7% Southeast Asian and 11% in the other category, so the comparison lacked power.

Educated cases, who graduated or completed some university or college were more likely than those who graduated or completed some high school, to report they would accept a nurse or technician visit to collect a blood sample in their home (OR = 2.15, 95% CI: 1.26–3.67). However, these women did not consistently indicate they would be more willing to provide other types of samples (blood in clinic, urine samples or saliva samples collected at home) or information to contact friends/colleagues to act as controls. No relationship between willingness to provide samples and reported income was observed. However, cases who “preferred not to answer” the annual income question were less willing than those in other income categories to indicate they would go to a clinic (52% vs. 59–75%), have a nurse visit (49% vs. 61–70%) or provide saliva (60% vs. 71–87%) or urine samples (morning urine: 55% vs. 65–79%; 24-hr urine 40% vs. 54–57%). Most of these differences were not significant.

None of the underweight cases (0%, n = 7 women) indicated they would go to a local clinic to give a blood sample. The percentage of normal weight, overweight, and obese cases who would attend a clinic was significantly higher (63–66%; p = 0.007) and a similar but non-significant pattern was observed for agreeing to have a nurse visit in the home (29% of underweight, 55–69% of normal, overweight and obese).

Finally, cases who consumed alcohol were significantly more likely to agree that they would provide contact information for friends. Only 1% of non-drinkers would provide this information, while 35% of moderate drinkers (1–6 drinks/week) and 32% of cases drinking 7 or more drinks per week (p = 0.048) agreed.

## Discussion

Increasingly, biological samples are collected in health studies and analyzed to provide estimates of exposures or to measure indicators of susceptibility or effect. Our study was designed to evaluate the willingness to provide these samples in women recently diagnosed with breast cancer and to evaluate whether younger women were more or less likely to agree to provide these samples.

Overall, the willingness to provide samples was very similar to the actual provision of blood that was observed in the past among breast cancer patients. At the Ontario site of the Cooperative Family Registry for Breast Cancer Studies (CFRBCS) 57% of normal risk and 63% of women diagnosed with breast cancer, who met criteria for increased genetic risk, provided in-clinic blood samples in 1996 [16]. Almost 15 years later in our study, 62% indicated they would agree to go to a local clinic to provide a blood sample. In studies conducted internationally, participation rates have been somewhat higher. In the Long Island Breast Cancer Study Project (LIBCSP) with cases recruited between 1996 and 1997, 73% of case and control subjects donated a blood sample after completing the questionnaire [10,11]. In the Shanghai Women’s Health Study (1996–2000) blood samples were obtained from 82% of cases and 84% of controls after completing an in-person interview [8,17]. Younger cases were somewhat less likely to indicate they would have a nurse or technician visit their home to collect a blood sample (63%) as compared to attending a local clinic (72%) and this difference was not apparent for older cases. This could be an indication of the willingness of younger cases to have unknown visitors in the home, their ability to travel to a clinic, their actual willingness to provide the sample or more likely, a combination of these factors.

Many cases in our study would also agree to provide a morning or a 24-hr urine sample and most would agree to provide saliva samples. These observations are consistent with what has been reported previously in Ontario. In one of our recent studies, 72% of 5000 breast cancer cases and population-based controls from Ontario provided saliva samples, and the response rates did not vary between case/control status [6,7]. Higher response rates were observed in a population-based breast cancer case control study in Wisconsin (2004–2005), where 90% of both cases and controls returned urine samples following completion of an interview. Similarly, 93% of cases and 83% of controls donated a spot urine sample in LIBCSP [10,11].

In our study, younger cases consistently indicated they were more likely to provide any type of biological sample, although these differences were generally not significant. They were significantly more likely to agree to provide a morning urine sample and ship it back to a laboratory than the older cases, but not whole day urine samples (24 hours) or saliva samples. We do not have an explanation for the increased willingness over older cases to provide a urine spot sample. We expected younger women to be less likely to agree due to time constraints which could include competing demands of children, aging parents, and full time employment during a cancer diagnosis. It is possible that younger women may be more motivated overall to provide biological samples in a search for the cause of their disease [10,11].

Cases who were underweight were less likely to agree to provide biological samples. Since self-reported pre-diagnosis weight (one year ago) was used to calculate BMI, it is unlikely that the low BMI is an indicator of more advanced disease, which could in part explain a lower response rate. In the LIBCSP, investigators found that increasing age and past smoking decreased likelihood that a respondent would donate blood. Subjects who identified as “white” or “other” and those who used alcohol were more likely to provide blood samples [11]. Similarly, in the Ontario CFRBCS, non- whites were less likely to provide blood samples (35% vs. 60% in white women and men with breast cancer) [16]. In our study, cases who drank alcohol (moderate or heavier use) were more likely to agree that they would provide contact information of friends/colleagues but were not more likely to agree to provide biological samples.

Overall, it is likely that female subjects enrolled in case-control studies of earlier onset breast cancer will agree to provide samples at slightly higher rates than those reported here. Our questions were hypothetical. In an etiologic study, following completion of dietary and other environmental questionnaires, subjects may be more interested and motivated to provide samples once an understanding of the study, its methods, and the investigators is gained. However, it is important to note that the potential participation/response rates were calculated using the “participants of the survey” as the denominator. Our actual response rate based on the population-based OCR would be lower since of the 462 eligible women identified by the OCR, 45 (9.7%) opted out and of the 417 remaining only 67% returned the completed questionnaires. Thus, if the total 462 eligible cases are used as the denominator (instead of 278), the overall response rates would be approximately half of those reported in Table 2. For example, only 35% of the cases would “go to a local clinic to give a blood sample” if the total eligible sample is used. In case-control studies of cancer that collect biological samples, we would only invite those women who consent to participate in the research and also fully complete questionnaire information on risk factors and important confounders. Thus, our expectation for overall response rates for biological sample collection from eligible cases are lower than those reported in Table 2.

Finally, in earlier focus group work we conducted in young women free of cancer, all indicated they would enroll in a study if a friend or a work colleague asked them and they would provide blood and/or urine samples [17]. These results were encouraging and we proposed to evaluate this method in cases with breast cancer to determine the feasibility for future studies, particularly the OEHS. We did not expect to find such low percentages of women (26%) willing to provide contact information for potential controls. This did not vary by education, race or age, but did vary by alcohol consumption and resulted in a decision to select an alternate method for the OEHS. It is possible that cases view their diagnosis as private, may not want to burden their friends, or have friends who are unaware of their diagnosis since we contacted them within 4 weeks of receiving a pathology report. It is likely that alternate methods of control recruitment, such as using social networking sites, may be more feasible in these younger populations.

In conclusion, the results of our study indicate that reasonable response rates for biological sample collection could be expected in future case-control studies of breast cancer in both younger and older women, but that other methods to select controls must be devised.
